# A Comparative Analysis and Limited Phylogenetic Implications of Mitogenomes in Infraorder-Level Diptera

**DOI:** 10.3390/ijms26157222

**Published:** 2025-07-25

**Authors:** Huan Yuan, Bin Chen

**Affiliations:** Chongqing Key Laboratory of Vector Control and Utinization, Institute of Entomology and Molecular Biology, College of Life Sciences, Chongqing Normal University, Chongqing 401331, China; 2022010513007@stu.cqnu.edu.cn

**Keywords:** Diptera, mitogenomes, phylogeny, evolution

## Abstract

Diptera comprises more than 154,000 described species, representing approximately 10–12% of insects. Members have successfully colonized all continents and a wide range of habitats. However, higher-level phylogenetic relationships within Diptera have remained ambiguous. Mitochondrial genomes (mitogenomes) have been used as valuable molecular markers for resolving phylogenetic issues. To explore the effect of such markers in solving the higher-level phylogenetic relationship of Diptera, we sequenced and annotated the mitogenomes of 25 species, combined with 180 mitogenomes from 33 superfamilies of dipteran insects to conduct a phylogenetic analysis based on the PCGsrRNA and PCGs12rRNA datasets using IQ-TREE under the partition model. The phylogenetic analysis failed to recover the monophyly of the two suborders Nematocera and Brachycera. Two of six infraorders within the Nematocera—Tipulomorpha and Ptychopteromorpha—were monophyletic. The ancestral Deuterophlebiidae were a strongly supported sister group of all remaining Diptera, but Anisopodidae, as the closest relative of Brachycera, received only weak support. Three of four infraorders within Branchycera—Tabanomorpha, Xylophagomorpha, and Stratiomyomorpha—were, respectively, supported as a monophyletic clade, except Muscomorpha due to the strong long-branch attraction between Cecidomyiidae and Nycteribiidae. The inferred infraordinal relationships followed the topology Tabanomorpha + (Xylophagomorpha + (Stratiomyomorpha + Muscomorpha)). However, the proposed topology lacks strong statistical support, suggesting alternative relationships remain plausible. Based on mitogenome data alone, we infer that Diptera originated earlier than the Late Triassic at 223.43 Mya (95% highest posterior density [HPD] 166.60–272.02 Mya) and the earliest brachyeran Diptera originated in the mid-Jurassic (171.61 Mya).

## 1. Introduction

The order Diptera (flies) is one of the four most species-rich orders, with more than 154,000 known species representing 10–12% of all insect diversity, colonizing all continents and nearly all habitats. This order comprises numerous ecologically and medically important groups, including mosquitoes, black flies, midges, fruit flies, blow flies, and house flies. Certain dipteran species, particularly hematophagous groups such as mosquitoes, pose substantial threats to human and animal health through the transmission of pathogens responsible for deadly diseases such as malaria, dengue fever, yellow fever, Zika virus, encephalitis, and filariasis [1,2]. Conversely, many dipterans provide essential ecosystem services, functioning as pollinators and biological control agents in both managed and natural ecosystems [3,4].

**Figure 1:**
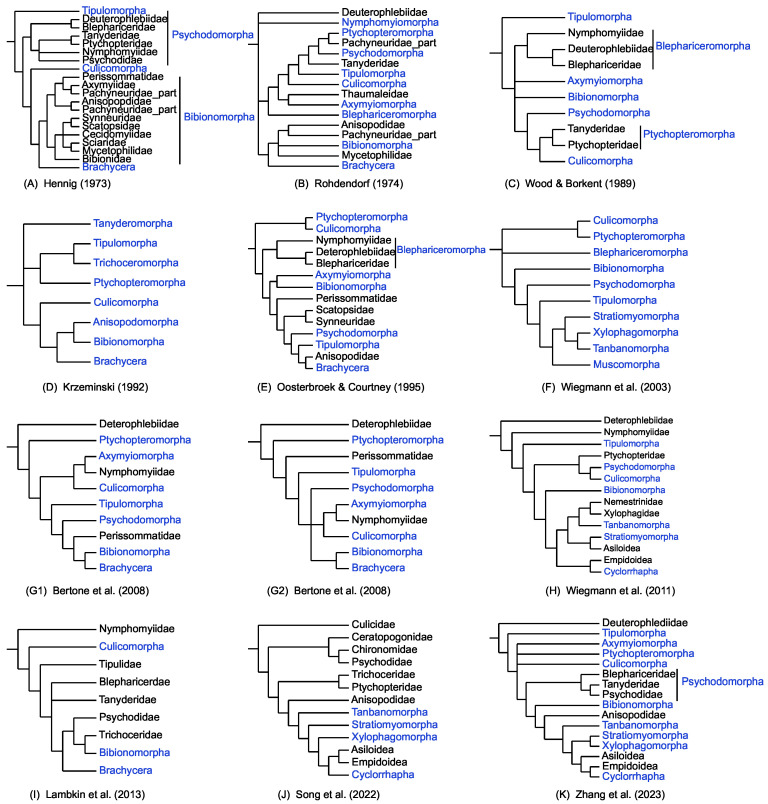
Phylogenetic hypotheses of higher-level Diptera from prior studies. (**A**) Phylogenetic hypothesis of lower Diptera relationships based on imaginal characters [9]. (**B**) Phylogenetic relationships of nematocerous families inferred from their morphological features [20]. (**C**) Cladogram showing the relationships between the families of Nematocera based on morphological characters [19]. (**D**) Phylogenetic relationships of the higher taxa of nematocerous Diptera [30]. (**E**) Cladogram of the families of nematocerous Diptera based on 98 morphological characters using parsimony-referred [24]. (**F**) Phylogeny of Brachycera based on combined data from 28S rDNA and morphology [22]. (**G1**) Parsimony analysis of combined nuclear ribosomal (28S) and protein-coding (CAD, PGD, and TPI) genes [13]. (**G2**) Majority rule consensus of Bayesian Markov chain Monte Carlo [13]. (**H**) Phylogenetic tree for Diptera based on molecular and morphological data [21]. (**I**) Bayes combined majority rule consensus tree [8]. (**J**) Phylogenetic relationships of Brachycera inferred from mitogenome [23]. (**K**) Bayesian tree of Nematocera based on mitogenome [12]. The blue fonts indicate taxa at the infraorder level or higher.

Contemporary dipteran taxonomy recognizes the order as comprising two suborders: Nematocera and Brachycera, 8–10 infraorders, 22–32 superfamilies, over 150 families, and approximately 10,000 genera, according to mainstream authorities [5,6,7,8]. Previous phylogenetic studies attempted to reveal the relationships of Diptera [9,10,11]. However, the number and composition of dipteran infraorders, particularly those within the suborder Nematocera, have been the subject of ongoing debate, which has subsequently led to the emergence of issues concerning their phylogenetic relationships. According to Hennig’s classification, Nematocera is traditionally divided into four infraorders: Tipulomorpha, Culicomorpha, Psychodomorpha and Bibionomorpha [9]. Recent studies have generally accepted six infraorders and some unplaced families, Tipulomorpha, Ptychopteromorpha, Culicomorpha, Axymyiomorpha, Psychodomorpha, Bibionomorpha, Deuterophlebiidae and Anisopodiidae [12,13] (Figure 1). Although, Tipulomorpha [14,15,16,17], Culicomorpha [18], Psychodomorpha and Bibionomorpha [8] as common infraorders are often discussed (Figure 1). The infraorder Blephariceromorpha has also been rarely mentioned in other works [11,19]. Classifications have proposed the existence of four to seven infraorders within Nematocera. The inter-infraorder relationships are enigmatic. Moreover, the families Deuterophlebiidae and Nymphomyiidae, which are the earliest extant fly lineages, have long been controversial [8,13,20,21]. Brachycera, characterized by its short antennae, is strongly supported as a monophyletic by congruent morphological, nuclear, and mitochondrial evidence [21], while the closest relative of this monophyletic suborder remains enigmatic. The nematoceran infraorder Bibionomorpha [9,13,21], Tipulomorpha [22], and the unusual and rarely collected family Anisopodidae [12,23,24] ever disappeared as the closest group of Brachycera. Brchycera can be classified into four infraorders: Xylophagomorpha containing the single family Xylophagidae; Tabanomorpha (8 families, including horseflies, snipeflies, and relatives); Stratiomyomorpha (3 families, including soldier flies and relatives); and Muscomorpha (100+ families: comprising all remaining Brachycera) [22,25]. The former three infraorders are occasionally recognized as a monophyletic SXT clade (Stratiomyomorpha + (Xylophagomorpha + Tabanomorpha)) in recent molecular phylogenies [22,25,26]; however, this clade typically exhibits low to moderate support. It is important to note that this clade is not consistently endorsed across all research studies [12,21,23], with the comprehensive phylogenetic analysis of Diptera serving as a notable exception [21]. The location of Asiloidea and Empiodoidea is another outstanding hypothesis that has been challenged by molecular data [12,21,23]. In brief, the monophyletic status and relationships among Brachyceran infraorders, as well as their lower taxa, remain largely contentious. The earliest dipteran fossils were discovered in the Upper Triassic of the Mesozoic era, approximately 240 million years ago [27,28]. The most comprehensive dating analyses revealed that Diptera originated in the Late Permian (250 Ma), preceding the earliest fossil evidence by approximately 20 million years [21]. Some fossil records for lower Brachycera, including crucial flower-visiting flies, have revealed co-evolutionary radiation with basal angiosperms, suggesting that lower Brachycera flourished in the middle of the Cretaceous period [29]. However, a molecular timescale for Brachycera hypothesized that Brachycera originated in the Late Triassic or the earliest Mesozoic and that all major lower brachyceran fly lineages had nearly contemporaneous origins in the mid-Jurassic period prior to the emergence of flowering plants [22]. Therefore, the geological epochs associated with the emergence of the majority of dipteran taxa have not yet been conclusively determined.

Mitochondria are vital organelles in eukaryotic cells and are involved in oxidative phosphorylation [31]. Conservation, easy alignment, maternal inheritance, and straightforward gene orthologs of the mitochondrial genome (mitogenome) have made it a significant source of molecular markers for evolutionary and phylogenetic studies [32]. Since 2004, there has been a significant increase in the number of insect species whose mitogenomes have been sequenced annually [31]. Up to December 2023, a total of 686 nearly complete mitogenomes of Diptera have been sequenced. Mitogenomes have proven effective for inferring intraordinal phylogenetic relationships across insects, with most results broadly congruent with other data sources [31,33,34]. Mitochondrial phylogenomic studies on Hemiptera [35] and Hymenoptera [36,37,38] have recovered relationships largely consistent with those inferred from nuclear data. In Coleoptera, mitogenome analyses using Archostemata as the outgroup have improved phylogenetic resolution [39]. Studies using mitogenomes from Lepidoptera have also produced highly congruent conclusions [40]. However, using only the mitogenome in phylogenetics faces challenges including gene content limitations, rate heterogeneity among lineages, and saturation effects for deep divergences, as documented in [41,42]. Notwithstanding these constraints, work by Cameron et al. [43] suggested that whole mitogenome analyses have the ability to resolve relationships over broad timescales with high precision in Diptera, providing valuable evidence for the resurrected Orthorrhapha and demonstrating the potential of mitogenome as a data source for deep-level studies. To systematically evaluate mitogenomic efficacy for infraorder-level dipteran phylogeny, we conducted the present study.

In this study, we sequenced and annotated 25 mitogenomes of dipteran insects from 9 superfamilies, 16 families, a combination of 180 published mitogenomes from 33 superfamilies, and 73 families of dipteran insects available in the GenBank database as ingroups to conduct phylogenetic analysis based on the PCGsrRNA and PCGs12rRNA datasets using IQ-TREE under the partition model. Additionally, we inferred the divergence time of the major phylogenetic nodes by incorporating nine fossil records as references. This study presents a comprehensive analysis of dipteran phylogenetics and the construction of an evolutionary timescale based on mitogenome data. By expanding our understanding of dipteran mitogenomics, our research contributes to resolving some previously debated phylogenetic issues within the Diptera order.

## 2. Results

### 2.1. General Features of 25 Mitogenomes

**Figure 2:**
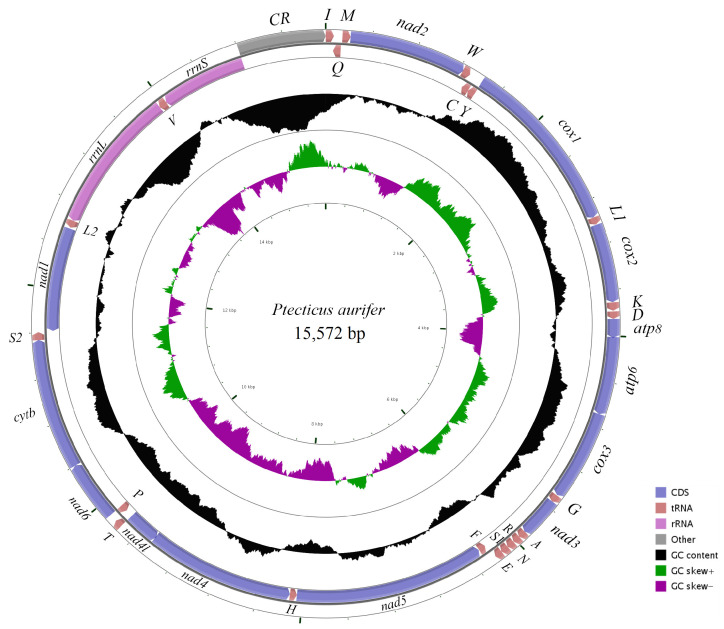
Circular map of the complete *P. aurifer* mitochondrial genome. Arrows indicate the direction of gene transcription. The GC content and GC-skew were plotted as the deviations from the average GC content and GC-shew of the entire sequence, respectively.

The set of 25 recently sequenced complete mitogenomes exhibits the characteristic features commonly observed in other published dipteran mitogenomes. These mitogenomes possess a circular structure and are highly compact, showing relatively conserved gene content. A total of 37 genes consisted of 13 protein-coding genes (PCGs), 22 transfer RNA (tRNA) genes, two ribosomal RNA (rRNA) genes, and an AT-rich region known as the control region (CR), which involves the origin of DNA replication. Among the 13 PCGs, nine were located on the majority strand (J-strand), whereas the remaining four PCGs, along with eight tRNAs and two rRNAs, reside on the minority strand (N-strand). The gene order and orientation in these mitogenomes were consistent with the putative ancestral insect arrangement. Notably, no gene rearrangements were observed, and the gene order closely resembled that of the common species *Ptectius aurifer* (Stratiomyidae), as determined by our analysis (Figure 2).

**Figure 3:**
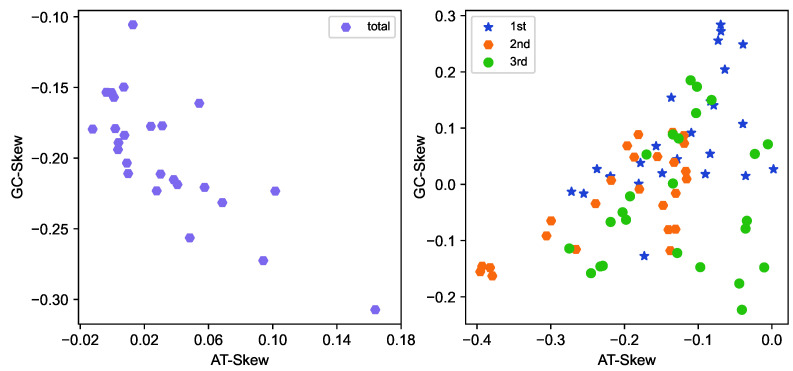
Nucleotide usage bias of the 25 newly sequenced mitogenomes, showing as AT-Skew = [A − T] / [A + T] and GC-Skew = [G − C] / [G + C]. (**Left**): All sites altogether; (**Right**): codon positions (1st/2nd/3rd) in PCGs. The start and stop codons are excluded.

The 25 newly sequenced mitogenomes exhibited a range of full-length sizes from 15,307 bp (*Loxoneura* sp.) to 16,585 bp (*Systropus daiyunshanus*), which falls within the typical range observed in other sequenced dipterans (14–20 kb). The variations in total length primarily resulted from differences in the size of the control region. A comparative analysis of the A + T content across the sequenced mitogenomes revealed a range of 69.9% (*Clephydroneura* sp.) and 81.1% (*Asarkina porcina*), and all nucleotide compositions showed a very strong bias (A + T)% > (G + C)% (Table 1). However, there are a few exceptions to this pattern. *Asarkina porcina* (Syrphidae), *Atylotus sinensis* (Tabanidae), *Spaniocelyphus* sp. (Celyphidae) showed slightly negative AT-Skew values (−0.0037, −0.0004, and −0.0122, respectively). Overall, the newly sequenced mitogenomes displayed a positive AT-Skew and negative GC-Skew, indicating a higher abundance of adenine (A) and cytosine (C) nucleotides (Figure 3).

### 2.2. Phylogenetic Inference

From the perspective of two phylogenetic results (Figures S2–S5), the previously controversial issues have been partially resolved, while the monophyly of many infraorders remains unconfirmed. The two topologyies were congruent with the traditional division of the monophyletic Diptera into two suborders: Nematocera and Brachycera. Nematocera, the older lineage, which at the base of Diptera, was confirmed to be paraphyletic. The highly specialized family Deuterophlebiidae, which had limited representation in our analysis, was the sister group to the remaining Diptera. The limited taxonomic sampling (single representative each) of Axymyiidae, Tanyderidae, Blephariceridae, Nymphomyiidae, and Anisopodidae in our phylogenetic analysis may affect the precision of their nodal placements. Consequently, these relationships were excluded from our definitive phylogenetic conclusions. The remaining nematocerous clades were classified into Tipulomorpha, Ptychopteromorpha, Culicomorpha, Psychodomorpha, and Bibionomorpha. Among these, only Tipulomorpha and Ptychopteromorpha were strongly supported as monophyletic clade and identified as the most basal infraorders. Tipuloidea (Tipulidae, Limoniidae and Cylindrotomidae) were strongly supported as sister groups (SH-aLRT/aBayes/UFBoot = 99.1/1/95). Ptychopteridae appears to be the sole family in Ptychopteromorpha. The Culicomorpha phylogeny showed discordance with traditional classification, forming two novel clades rather than the expected Culicoidea and Chironomoidea superfamilies. In the two novel clades, only the Corethrellidae-Culicidae (100/1/100) and Thaumaleidae-Simuliidae (100/1/100) sister groups were recovered and strongly supported. Between the two culicomorphan clades, Bibionomorpha is a polytomy consisting of Scatopsidae, Cecidomyiidae and a clade composed of [(Pachyneuridae + (Pleciidae + Bibionidae)) + (Keroplatidae + (Mycetophilidae + Sciaridae))] (100/1/100). The monophyletic groupings of (Blephariceridae + Tanyderidae) (97.5/1/90) and Axymyiidae were suggested but not strongly supported.

Brachycera, a higher-level lineage, was not supported as a monophyletic group due to the nesting of Cecidomyiidae (Figures S3 and S5). Four infraorders were identified within Brachycera, which had long been recognized and were strongly supported: Tabanomorpha, Xylophagomorpha, Stratiomyomorpha, and Muscomorpha. The relationship between the four infraorders is (Tabanomorpha + (Xylophagomorpha + (Stratiomyomorpha + Muscomorpha)), and the infraorders Tabanomorpha, Xylophagomorpha and Stratiomyomorpha, are located at the base of Branchycera. However, there are differences in the familial relationships of these infraorders between the two datasets. The concordance factors of the PCGs12RNA dataset were shown to be slightly better than those of the PCGsRNA dataset (Figures S6 and S7). Consequently, further interpretations of the tree would be based on it. Tabanomorpha (SH-aLRT/aBayes/UFBoot = 99.5/1/99), represented by the Tabanoidea superfamily, formed a monophyletic basal lineage within Brachycera. This clade was the closest relative of Anisopodidae, which were the higher-level nematocerans. Xylophagomorpha, represented by the sole family Xylophagidae, formed a clade with Nemestrinidae, but with moderate support. While Stratiomyomorpha, represented by Stratiomyidae, was strongly supported as monophyletic (100/1/100). Muscomorpha was not recovered as monophyletic in our analyses due to long-branch attraction (LBA) between Cecidomyiidae (Sciaroidea) and Nycteribiidae (Hippoboscoidea) (Table S3). Notably, Cecidomyiidae was displaced from its traditional position in Nematocera but was also not correctly grouped within Muscomorpha.

The Muscomorpha includes 17 superfamilies: Asiloidea, Empidoidea, Platypezoidea, Syrphoidea, Conopoidea, Sciomyzoidea, Ephydroidea, Neriodiea, Lauxanioidea, Sphaeroceroidea, Tephritoidea, Diopsoidea, Carnoidea, Opomyzoidea, Hippoboscoidea, Oestroidea, Muscoidea. With the exception of Syrphoidea, Opomyzoidea, Sciomyzoidea, Lauxanioidea, and Diopsoidea, which were supported as polyphyletic groups, and Oestroidea, which was found to be paraphyletic, the remaining eleven superfamilies have been confirmed as monophyletic. The superfamily Asiloidea was strongly supported as the basal lineage of Muscomorpha (99.8/1/96) and sister to Eremoneura, a clade comprising all remaining muscomorphan superfamilies. Eremoneura comprised two monophyletic subclades: Empidoidea and Cyclorrhapha (98.5/1/97), with their weak sister relationship. Cyclorrhapha comprised two traditional divisions: Aschiza (containing Platypezoidea and Syrphoidea) and Schizophora. Although both superfamilies were resolved as monophyletic, statistical support remained limited. Notably, Syrphoidea was unexpectedly recovered within Schizophora, rendering Aschiza non-monophyletic. The remaining thirteen superfamilies belong to Schizophora, with phylogenetic analyses supporting the paraphyly of Acalyptratae and monophyly of Calyptratae, consistent with prior studies. Among the ten Acalyptratae superfamilies analyzed, six (Conopoidea, Ephydroidea, Nerioidea, Sphaeroceroidea, Tephritoidea, and Carnoidea) were resolved as monophyletic, whereas Opomyzoidea, Sciomyzoidea, Lauxanioidea, and Diopsoidea were not, and the relationships between them did not receive strong support. We identified two alternative sister groups for Calyptratae: Opomyzoidea and Ephydroidea. In Calyptratae, long-branch attraction (LBA) artifacts were observed between Hippoboscoidea and Cecidomyiidae (Figure S3), complicating the assessment of Hippoboscoidea’s phylogenetic position and monophyly, but it did not affect that the muscoids and oestroids are always sister groups.

### 2.3. Divergence Time Estimation

**Figure 4:**
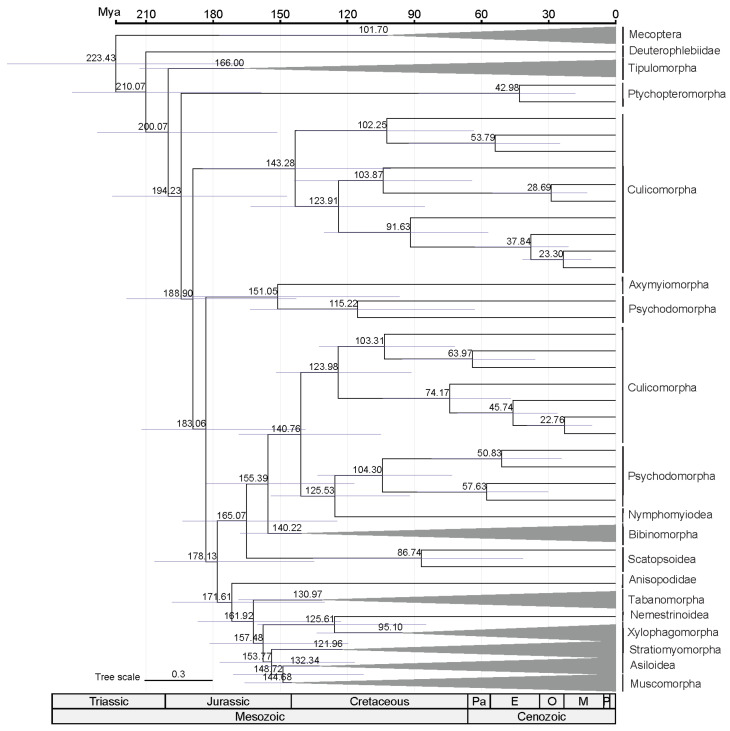
Divergence times of clades in Diptera estimated using MCMCTree based on nine fossil calibration points. Purple bars represent 95% credibility intervals for the clade ages. The numbers above the purple bars represent the divergence of the corresponding nodes. A geological time scale is shown at the bottom.

Divergence time estimation using MCMCTree for the PCGsRNA dataset provides insights into the evolutionary history of flies (Figure 4). It is inferred that Diptera diverged at 223.43 Mya (95% highest posterior density [HPD] 166.60–272.02 Mya), approximately 22 million years before the Late Triassic boundary (201 Mya). The earliest nematoceran clade radiated at 210.07 Mya (95% [HPD] 158.47–242.84 Mya). Later, the brachyceran infraorders diverged sequentially between 171.61 Mya (95% HPD 130.05–198.36 Mya, divergence of Tabanomorpha and the rest of Brachycera) and 148.72 Mya (95% HPD 112.76–170.86 Mya, Muscomorpha). Muscomorpha subsequently underwent rapid diversification during the Early Cretaceous.

## 3. Discussion

From the perspective of the two phylogenetic results, the previously controversial issues have been partially resolved, while the monophyly of many infraorders remains unconfirmed. Both datasets recovered the congruent arrangements of six infraorders within Nematocera. This conflicts with morphological hypotheses placing Tipulomorpha as sister to all other Diptera [9,19,27,44]. Deuterophlebiidae is recognized as the earliest extant fly and a sister group to all remaining Diptera, consistent with some authors’ acceptance [12,13,21]. Tipulomorpha (Trichoceridae + Tipuloidea) was resolved as monophyletic, consistent with established morphological and molecular evidence [15,16,24]. This conflicts with Wood and Borkent’s concept of Tipulomorpha being restricted to Tipuloidea, and Trichoceridae assigned to Psychodomorpha [19]. Traditional Ptychopteromorpha comprised Ptychopteridae and Tanyderidae [19,24,45]. However, our phylogenetic analysis revealed that Ptychopteridae was the sole family of Ptychopteromorpha, and Tanyderidae was sister to Blephariceridae within Psychodomorpha. Blephariceridae together with Deuterophlebiidae and Nymphomyiidae were previously categorized within Blephariceromorpha [19,24], a now-defunct group [12]. Our analyses did not recover monophyly for Culicomorpha, as it formed two distinct lineages but not the Culicoidea and Chironomoidea typically recognized [12,13,18,46]. Axymyiidae was recognized as the sole family within the monotypic Axymyiomorpha because of the absence of synapomorphies with other flies [19]. In our analyses, Axymyiomorpha and members of Psychodomorpha (Tanyderidae + Blephariceridae) formed a clade with low support, suggesting that the Axymyiomorpha location may be ambiguous. The psychodomorphan members are highly mobile. Hennig’s system included families Deuterophlebiidae, Blephariceridae, Tanyderidae, Ptychopteridae, Nymphomyiidae, and Psychodidae [9]. Later classifications by Krivosheina (1988) and Wood and Borkent (1989) expanded this concept to include Anisopodidae, Canthyloscelidae, Perissommatidae, Scatopsidae, and Trichoceridae, reflecting conflicting interpretations of morphological synapomorphies [19]. While recent molecular studies recover Blephariceridae, Tanyderidae, and Psychodidaewithin Psychodomorpha [12,13,21]. However, both Lambkin et al. [8] and our analyses reject this grouping. Bibionomorpha traditionally comprises seven families: Cecidomyiidae, Sciaridae, Mycetophilidae, Keroplatidae, Pleciidae, Bibionidae, and Pachyneuridae. Our analyses revealed significant long-branch attraction (LBA) between Cecidomyiidae (Bibionomorpha) and Nycteribiidae (Hippoboscoidea), attributable to their accelerated evolutionary rates. This artifact resulted in the anomalous clustering of Cecidomyiidae with Nycteribiidae, rather than with other bibionomorph families. Although Scatopsidae was previously placed in Bibionomorpha [10,12,13,21], our results exclude it from Bibionomorpha. The sister group to Brachycera within Nematocera remains contentious, as the arrangement of the lower dipteran infraorders remains unresolved. In our analyses, Anisopodidae was weakly supported as the sister group to Brachycera, a relationship consistent with mitogenomic studies [12,23]. This conflicts with alternative hypotheses placing Bibionomorpha as the closest brachyceran relative, which is supported by nuclear gene analyses [13] and combined morphological-molecular datasets [21]. The persistent incongruence among studies underscores the ongoing uncertainty regarding deep nematoceran relationships, particularly concerning the placement of these infraorders.

Three distinct topological hypotheses have been proposed for the relationships among the four brachyceran infraorders. Yeates et al. recovered the topology ((Stratiomyomorpha + (Xylophagomorpha + Tabanomorpha)) + Muscomorpha) based on 101 discrete morphological characters, collectively referring to the (Stratiomyomorpha + (Xylophagomorpha + Tabanomorpha)) grouping as the ‘SXT’ clade [25]. Although this hypothesis has been proposed in subsequent studies, it is not difficult to find that its branches lack credible support [22,26], and other studies failed to recover the SXT clade in independent analyses [21,23]. In contrast, Song et al. employed mitogenomes and proposed an alternative arrangement: (Tabanomorpha +(Stratiomyomorpha + (Xylophagomorpha + Muscomorpha))). Notably, their study did not support the SXT grouping and reported weak support for key nodes. Our phylogenetic analyses propose a new topology for brachyceran infraorders: (Tabanomorpha + (Xylophagomorpha + (Stratiomyomorpha + Muscomorpha))). This hypothesis is distinguished by strong support values at each node, with the exception of the node corresponding to (Muscomorpha + Stratiomyomorpha). Numerous studies demonstrate the lack of a consensus on this matter, indicating that the placement of these branches within Brachycera remains unresolved. While mitochondrial genomes provide a certain degree of phylogenetic signals, our study has inherent limitations due to the use of mtDNA alone. Potential biases may arise from factors such as accelerated evolutionary rates in Diptera, compositional heterogeneity, or incomplete lineage sorting. These results should therefore be considered hypothetical until corroborated by multi-locus nuclear data. Future studies combining universal single-copy orthologs, ultraconserved elements or transcriptomes would help validate our findings.

Divergence time estimation results indicated that Diptera diverged earlier than the Late Triassic period. This estimate aligns with the phylogenetic evidence from Blagoderov et al. [27] and deposits’ age described by Krzeminski et al. [47], but it is significantly younger than previous estimates by Wiegmann et al. [21]. The most abundant dipteran fossils were collected in Triassic entomofauna [48], and coincided with the post-Permian biotic recovery following the end-Permian mass extinction. Our results suggest that the earliest brachyceran Diptera originated in the mid-Jurassic (171.61 Mya) with the fossil *Oligophryne fungivoroides* (195–201.3 Mya) providing a minimum age constraint. Multiple flower-visiting families (e.g., Nemestrinidae, Stratiomyidae, Empididae, Syrphidae) subsequently radiated during the Late Cretaceous. Phylogenetic evidence supports the hypothesis that angiosperm-pollinator coevolution served as a key driver of diversification in both groups [49].

## 4. Materials and Methods

### 4.1. Sample Collection and DNA Extraction

Newly sequenced samples in this work were collected from valleys in Chengkou County, Chongqing Province, from July to August 2017 (Table 1). Twenty-five species from 18 families were identified based on their morphological characteristics and subsequently stored in 95–100% ethanol, at −20 °C until DNA extraction at the Institute of Entomology and Molecular Biology, College of Life Sciences, Chongqing Normal University, Chongqing, China. A total of 0.2 μg, and 40 ng/μL genomic DNA per sample was extracted from the thoracic muscle tissues using the Qiagen Genomic DNA Kit (Qiagen, Duesseldorf, Germany) at the Solution Limited (SZHT) Institute (Shenzhen, China).

### 4.2. Sequencing, Assembly, Annotation and Compositional Analysis

High-throughput sequencing was performed using the Illumina HiSeq X10 sequencing platform. A 350 bp paired-end library was generated using the DNA Library Prep Kit for Illumina (Cali., USA). Following the manufacturer’s recommendations, index codes were added to achieve a sequencing depth of 100×, and over 10 Gb of raw data were generated for each sample. High-quality reads were gained after removing adapters and poly-N sequences, and reads with more than 50% low quality bases (Q ≤ 5) using FastQC (http://www.bioinformatics.babraham.ac.uk/projects/fastqc/, accessed on 21 February 2019), and clean mitogenomic reads were extracted by Basic Local Alignment Search Tool (https://blast.ncbi.nlm.nih.gov/Blast.cgi, accessed on 21 February 2019) against the read pool with known relative sequences as query sequences. Clean reads were assembled into contigs using the de novo assembler SPAdes v. 3.9.0 [50]. The contigs were further connected to a complete mitogenome using NOVOPlasty v. 2.6.2 [51]. Subsequently, we manually corrected the annotations of 13 protein-coding genes (PCGs) and two rRNA genes using Geneious v. 9.0.2 [52], and the nucleotide composition and gene organization statistics were calculated simultaneously. Open reading frames (ORFs) prediction was also performed on the sequences using the invertebrate mitochondrial codon table, and the predicted ORFs were compared with those of other related species using the blastp and nr databases. The tRNA genes were submitted to tRNAscan-SE (http://lowelab.ucsc.edu/tRNAscan-SE/, accessed on 30 May 2019) to correct and predict the secondary structure. After annotation, the complete mitogenome was cyclized using the CGView Server (http://stothard.afns.ualberta.ca/cgview_server/, accessed on 7 June 2019) [53]. The complete mitogenome was edited using Sequin (https://www.ncbi.nlm.nih.gov/genbank/htgs/sequininfo/, accessed on 4 May 2020) to generate a submission that could be submitted to GenBank. The formalas AT-skew = [(A − T) / (A + T)] and GC-skew = [(G − C) / (G + C)] of the newly sequenced mitogenomes were calculated to investigate nucleotide composition bias [54,55], and scatterplots of AT-Skew, GC-Skew, and AT% were drawn using Python v. 3.6.8.

### 4.3. Sequence Retrieval, Matrix Generation and Bioinformatic Analysis

For phylogenetic analyses, we selected taxa from as many recognized families of Diptera as possible. Our phylogenetic analysis included 25 newly sequenced and 180 published sequences from the GenBank database, representing 89 families and 33 superfamilies as ingroups. Furthermore, we used two mecopterans (*Boreus elegans* and *Neopanorpa pulchra*) as outgroups (all sequences were downloaded as of 30 May 2023) (Table S1).

Multiple sequence alignment precedes matrix generation. The PCGs and rRNAs of 207 species were extracted by Geneious v. 9.0.2, and then the PCGs were aligned by the codon-aware program MACSE v. 2.06 and two rRNAs were aligned by MAFFT v. 7 with the G-INS-i strategy [56]. Subsequently, the 13 PCG alignments were trimmed using Gblocks under the invertebrate mitochondrial genetic code [57], while the 2 rRNA alignments underwent trimming using trimAl v. 1.2rev57. Finally, all individual alignments were concatenated into a supermatrix using the Phylosuite v. 1.2.3 platform with default settings [58,59]. Two matrices were generated prior to the phylogenetic inference. (1) PCGsrRNA, 13 PCGs plus two rRNA genes resulting in 12,426 nucleotides length; (2) PCGs12RNA, to mitigate substitution saturation, the third codon positions of 13 PCGs were removed, then plus two rRNA genes resulting in a 8859 nucleotide length.

Before phylogenetic analysis, the selection pressure of the 13 PCGs of the ingroups was analyzed by calculating the Ka (non-synonymous mutation rates) and Ks (synonymous mutation rates) values using DnaSP v. 6.11.1 [60]. The assessment of substitution saturation for each codon position within all PCGs was performed using the index (Iss) as implemented in DAMBE v. 7.2.102 [61]. The completeness of multiple sequence alignments was quantified by AliStat v. 1.11 [62], and the heterogeneity of the sequence was visualized using Aligroove v. 1.08 [63] before phylogenetic analyses (Figure S1).we have cited Figure S1 in the main part at the end of Section 4.3.

### 4.4. Phylogenetic Analyses and Tree Calibration

To determine the best partitioning schemes and corresponding nucleotide substitution models for each dataset, we employed ModelFinder for partitioned maximum likelihood analyses in IQ-TREE [64], and the Bayesian information criterion (BIC) and the ‘greedy’ algorithm were used, with branch lengths estimated as ‘unlinked’, to search for the best-fit scheme in the partition model. The support values of the inferred topologies were assessed by the Shimodaira–Hasegawa approximate likelihood ratio test (SH-aLRT > 80), Bayesian-like transformation of aLRT (aBayes > 0.8), and ultrafast bootstrap (UFBoot > 95) [65]. We quantified genealogical concordance with the gene concordance factor (gCF) and the site concordance factor (sCF) given the reference tree and gene trees [66].

To estimate the evolutionary ages of Diptera, nine fossil calibration points [21,28,67,68,69,70] were utilized as the node time prior for different grades (Table S2) using MCMCTree in PAML v. 4.9j under the GTR molecular clock model [71]. Here, the extinct species *Grauvogelia arzvilleriana* (238–241Ma) was considered to be the oldest known representative of Diptera [28,70] from the Paleobiology Database (https://paleobiodb.org/, accessed on 17 September 2023), so we placed this period prior to the Diptera crown. Hessian matrices were calculated using the GTR substitution model and the independent rate clock model. The preferred topology estimated from the partition maximum likelihood is selected as the input tree. We sampled every 10 iterations until 5,000 samples were gathered and set 20,000 iterations as burn-in. Additional details regarding the parameter settings, calibration points, and MCMC runs are available in the control file. The convergence of the MCMC runs was assessed based on convergence and infinite-site plots following the guidelines provided in the manual. Phylogenetic trees were visualized using the itol.toolkit v. 1.1.9 R package [72] and the iTOL web platform (https://itol.embl.de/, accessed on 19 October 2024).

## 5. Conclusions

A comparison of mitogenome sequences in the Diptera shows that mitogenomes reveal several general features. The gene composition and the number of 25 mitogenomes are identical to those of an ancestral diptera mitogenome, and the lengths of these mitogenomes are similar to the ancestral ones. Our phylogenetic analysis identified that the suborder Nematocera was paraphyletic and the Brachycera was non-monophyletic. Concerning the six infraorders within the Nematocera: Tipulomorpha, Ptychopteromorpha, Culicomorpha, Axymyiomorpha, Psychodomorpha, and Bibionomorpha, only the former two certainly recovered their monophyly. Deuterophlebiidae, as the earliest extant fly, were strongly recognized as a sister group of all remaining Diptera, and Anisopodidae as the closest relative of Branchycera, with little support. Three of four infraorders within Branchycera: Tabanomorpha, Xylophagomorpha, and Stratiomyomorpha, were supported as a monophyletic clade, except Muscomorpha was affected by the strong long-branch attraction between Cecidomyiidae and Nycteribiidae. The relationship between infraorder is Tabanomorpha + (Xylophagomorpha + (Stratiomyomorpha + Muscomorpha)). The evolution history of the flies resulting from MCMCTree based on the PCGs12RNA dataset reveals that Diptera originated earlier than the late Triassic at 223.43 Mya (95% [HPD] 166.60–272.02 Mya) and the earliest brachyeran Diptera originated in the mid-Jurassic (171.61 Mya). Although we attempted to account for the whole group evolution pattern of dipterans, we are still constrained by the challenges of adequately sampling the enormous number of species representing extant dipteran diversity. Families such as Ptychopteridae, Corethrellidae, Axymyiidae, Tanyderidae, Blephariceridae, Pachyneuridae, Anisopodidae, Nemestrinidae, Xylomyidae, Heleomyzidae, Chamaemyiidae and others that were not included in our study or had limited sampling pose a challenge to achieving a comprehensive phylogenetic interpretation. These inferred phylogenetic hypotheses include numerous unsupported nodes, indicating that mitochondrial genomic data alone are limited to confidently resolving higher-level relationships. Our phylogenetic reconstruction should be interpreted cautiously, as current limitations in both taxonomic sampling and molecular data constrain robust resolution. Future studies incorporating expanded taxon representation and multi-locus datasets will be critical for establishing more definitive phylogenetic relationships among these lineages.

## Figures and Tables

**Table 1:** Mitogenome information summary for 25 newly sequenced Diptera species.

Family	Species	Length (bp)	A Content (A%)	T Content (T%)	G Content (G%)	GC Content (GC%)	AT- Skew	GC- Skew	Accession No
Tipulidae	*Holorusia flava flava*	16,557	38.80	38.10	9.20	23.10	0.0091	−0.2035	MT511104
	*Antocha* sp.	15,578	40.00	35.90	10.10	24.10	0.0540	−0.1618	MT511110
Limoniidae	*Conosia irrorata*	15,374	40.20	37.80	8.70	22.00	0.0307	−0.2091	MT511115
	*Hexatoma* sp.	15,883	39.00	36.90	9.40	24.20	−0.0277	−0.2231	MT511103
Keroplatidae	Keroplatidae sp.	15,879	40.60	33.10	10.70	26.30	0.1018	−0.1863	MT511114
Stratiomyidae	*Ptecticus aurifer*	15,572	38.00	37.80	9.70	24.10	0.0026	−0.1950	MT511124
Asilidae	*Clephydroneura* sp.	15,714	40.70	29.30	10.40	30.00	0.1629	−0.3067	MT424762
	*Tabanus* sp.	15,991	39.20	38.90	8.90	22.00	0.0038	−0.1909	MT511107
Tabanidae	*Atylotus sinensis*	15,319	38.80	38.80	9.50	22.40	0.0000	−0.1518	MT511118
	Tabanidae sp.	15,648	40.10	35.50	9.40	24.40	0.0608	−0.2300	MT511116
	*Homoneura* sp.	16,208	38.60	38.00	9.60	23.50	0.0078	−0.1830	MT511108
Lauxaniidae	*Homoneura* sp.	16,284	38.90	38.30	9.70	22.80	0.0078	−0.1490	MT511111
	*Lauxaniidae* sp.	16,279	31.90	41.80	12.60	26.30	−0.1343	−0.0418	MT511112
Celyphidae	*Spaniocelyphus* sp.	15,342	37.60	38.50	9.80	23.90	−0.0118	−0.1799	MT511119
Syrphidae	*Phytomia zonata*	15,537	40.80	38.40	8.60	20.80	0.0303	−0.1731	MT511105
	*Asarkina porcina*	15,477	40.40	40.70	80.00	18.90	−0.0037	−0.1534	MT511106
	*Melanostoma* sp.	15,610	41.00	40.00	8.50	19.00	0.0123	−0.1053	MT511120
	*Microdon* sp.	15,770	42.00	38.10	7.40	19.90	0.0487	−0.2563	MT511101
Calliphoridae	*Chrysomya megacephala*	15,908	39.50	37.60	9.40	22.90	0.0246	−0.1790	MT511113
Sarcophagidae	*Blepharipa* sp.	15,835	41.40	38.40	7.90	20.20	0.0376	−0.2178	MT511109
Tachinidae	*Tachinidae* sp.	16,291	40.90	37.70	8.40	21.50	0.0407	−0.2186	MT511123
Tephritidae	*Zeugodacus depressus*	16,546	39.70	32.60	10.20	28.10	0.0982	−0.2740	MT477832
Platystomatidae	*Loxoneura* sp.	15,307	39.90	32.50	10.70	27.60	0.1022	−0.2246	MT511102
Empididae	*Hercostomus potanini*	15,633	38.70	34.50	10.40	26.70	0.0574	−0.2210	MT511125
Bombyliidae	*Systropus daiyunshanus*	16,585	39.60	34.50	9.90	25.80	0.0688	−0.2326	MT511117

## Data Availability

Data associated with this study are openly available from the National Center for Biotechnology Information at https://www.ncbi.nlm.nih.gov, (accession numbers: MT424762, MT477832, MT511101-MT511120, MT511123-MT511125, accessed on 30 May 2023). All data generated or analyzed during this study are included in this published article and its Supplementary Information Files.

## Supplementary Materials

The supporting information can be downloaded at: https://www.mdpi.com/article/10.3390/ijms26157222/s1.
