# Supplementary material for: A Comparative Analysis and Limited Phylogenetic Implications of Mitogenomes in Infraorder-Level Diptera
Source: Int J Mol Sci. 2025 Jul 25;26(15):7222. doi: 10.3390/ijms26157222 (PMC12346218; doi:10.3390/ijms26157222)
Supplement: Supplementary file 1 [file ijms-26-07222-s001.zip › Figure. S3 tree of PCGs12RNA.pdf]

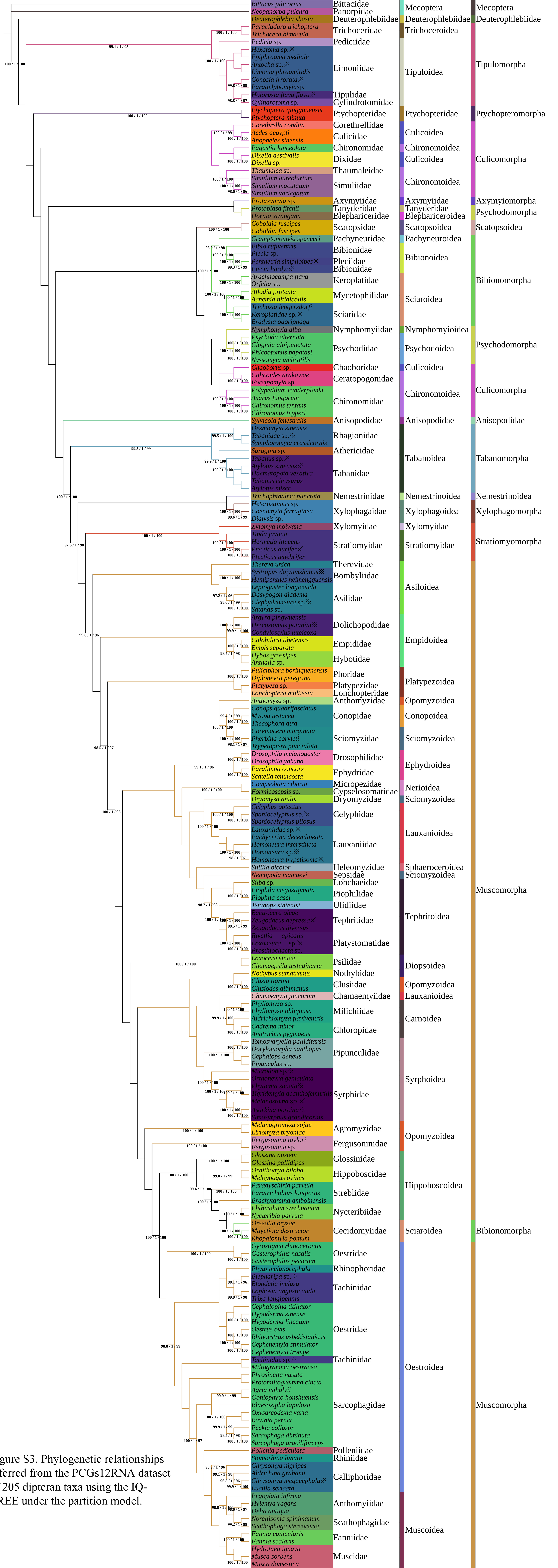

Figure S3. Phylogenetic relationships inferred from the PCGs12RNA dataset of 205 dipteran taxa using the IQ-TREE under the partition model.
