# Supplementary material for: A Comparative Analysis and Limited Phylogenetic Implications of Mitogenomes in Infraorder-Level Diptera
Source: Int J Mol Sci. 2025 Jul 25;26(15):7222. doi: 10.3390/ijms26157222 (PMC12346218; doi:10.3390/ijms26157222)
Supplement: Supplementary file 1 [file ijms-26-07222-s001.zip › ijms-3658081-Supplementary information.pdf]

***Yuan et al.* Supplementary Information**

Table of Contents

I. Supplementary Results and discussion 1
1. Compositional heterogeneity and completeness test for matrices 1
2. Concordance factor analysis for matrices 1
II. Supplementary Figures Legends..1
III. Supplementary Table Legends 2

I. Supplementary results and discussion

1. Compositional heterogeneity and completeness test for matrices

A heterogeneity test comparing the PCGsRNA and PCGs12RNA datasets indicated a high degree of similarity between the two overall. However, in the PCGsRNA dataset, several taxa were flagged with red highlights (Figure S1A). This suggests that the inclusion of the 3rd codon positions in the analysis introduced confounding signals, potentially due to substitution saturation detected by DAMBE. These outliers noticeably influenced certain individuals; however, we reconstructed the topology based on the PCGs12RNA dataset. Additionally, we assessed the completeness of the multiple sequence alignments in these datasets and found that the nucleotide alignments did not exhibit non-randomly distributed missing data (Figure S1B).

2. Concordance factor analysis for matrices

We conducted the concordance factors [the gene concordance factor (gCF) and the site concordance factor (sCF)] for every branch of the reference tree calculated by the partitional model in IQ-tree (Figures S4-5). The Concordance of PCGs12RNA dataset seems to be slightly better than that of PCGsRNA dataset as shown in the corresponding factor plots (Figures S6-7), it indicates that the topology based on PCGs12RNA dataset obtained more decisive genes and sites support.

II. Supplementary Figures Legends

Figure S1. Heterogeneity and completeness of PCGsRNA and PCGs12RNA dadasets for 205 species. (A) The mean similarity scores between sequences ranging from −1 in red to 1 in blue, calculated using Aligroove. (B) Completeness scores for pairs of sequences ranging from 0 to 1, calculated using AliStat. The upper right half plot indicates PCGsRNA, and the lower-left half for PCGs12RNA. Species are denoted on the Y-axis direction, and correspond to X-axis in folding symmetry.

Figure S2. Phylogenetic relationships inferred from the PCGsRNA dataset of 205 dipteran taxa using the IQ-TREE under the partition model. The numbers in the branches are SH-aLRT support (%)/aBayes support/ultrafast bootstrap support (%).

Figure S3. Phylogenetic relationships inferred from the PCGs12RNA dataset of 205 dipteran taxa using the IQ-TREE under the partition model. The numbers in the branches are SH-aLRT support (%)/aBayes support/ultrafast bootstrap support (%).

Figure S4. Phylogenetic relationships inferred from the PCGsRNA dataset of 205 dipteran taxa using the IQ-TREE under the partition model. The numbers in the branches are the ultrafast

bootstrap/gCF/sCF (%).

Figure S5. Phylogenetic relationships inferred from the PCGs12RNA dataset of 205 dipteran taxa using the IQ-TREE under the partition model. The numbers in the branches are the ultrafast bootstrap/gCF/sCF (%).

Figure S6. The concordance factors of the PCGsRNA dataset.

Figure S7. The concordance factors of the PCGs12RNA dataset.

III. Supplementary Table Legends

Table S1. A total of 205 Diptera mitogenomes were selected for comparative and phylogenetic analyses in this study.

Table S2. Calibration points, fossil information, calibration scheme, and references used in the analyses to estimate divergence dates.

Table S3. The long branch score calculation for PCGs12RNA dataset based on partition model are listed in descending order.
