# Supplementary figures and images for: A Comparative Analysis and Limited Phylogenetic Implications of Mitogenomes in Infraorder-Level Diptera
Source: Int J Mol Sci. 2025 Jul 25;26(15):7222. doi: 10.3390/ijms26157222 (PMC12346218; doi:10.3390/ijms26157222)

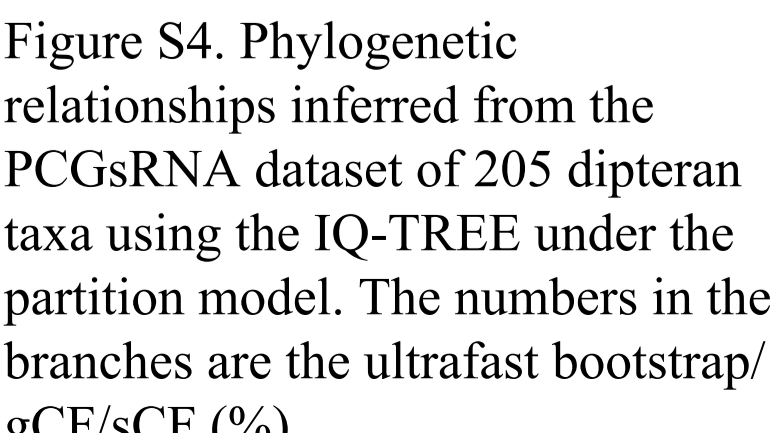

Supplement: Supplementary file 1 [file ijms-26-07222-s001.zip › Figure. S4 concordance_PCGsRNA.pdf]

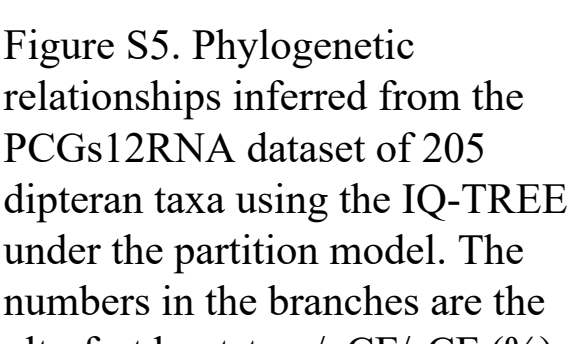

Supplement: Supplementary file 1 [file ijms-26-07222-s001.zip › Figure. S5 concordance_PCGs12RNA.pdf]

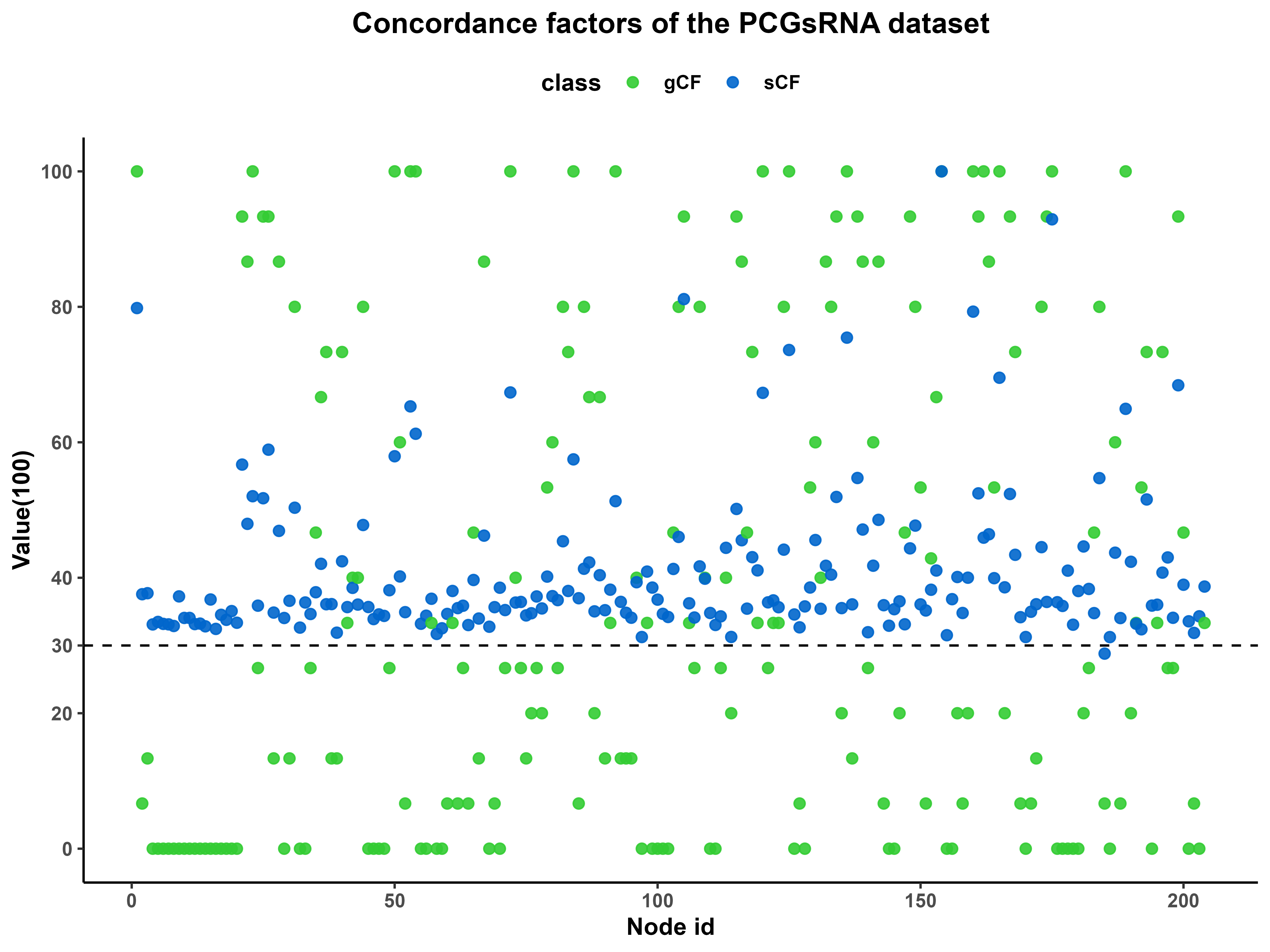

Supplement: Supplementary file 1 [file ijms-26-07222-s001.zip › Figure. S6 concord_PCGsRNA_plot.png]

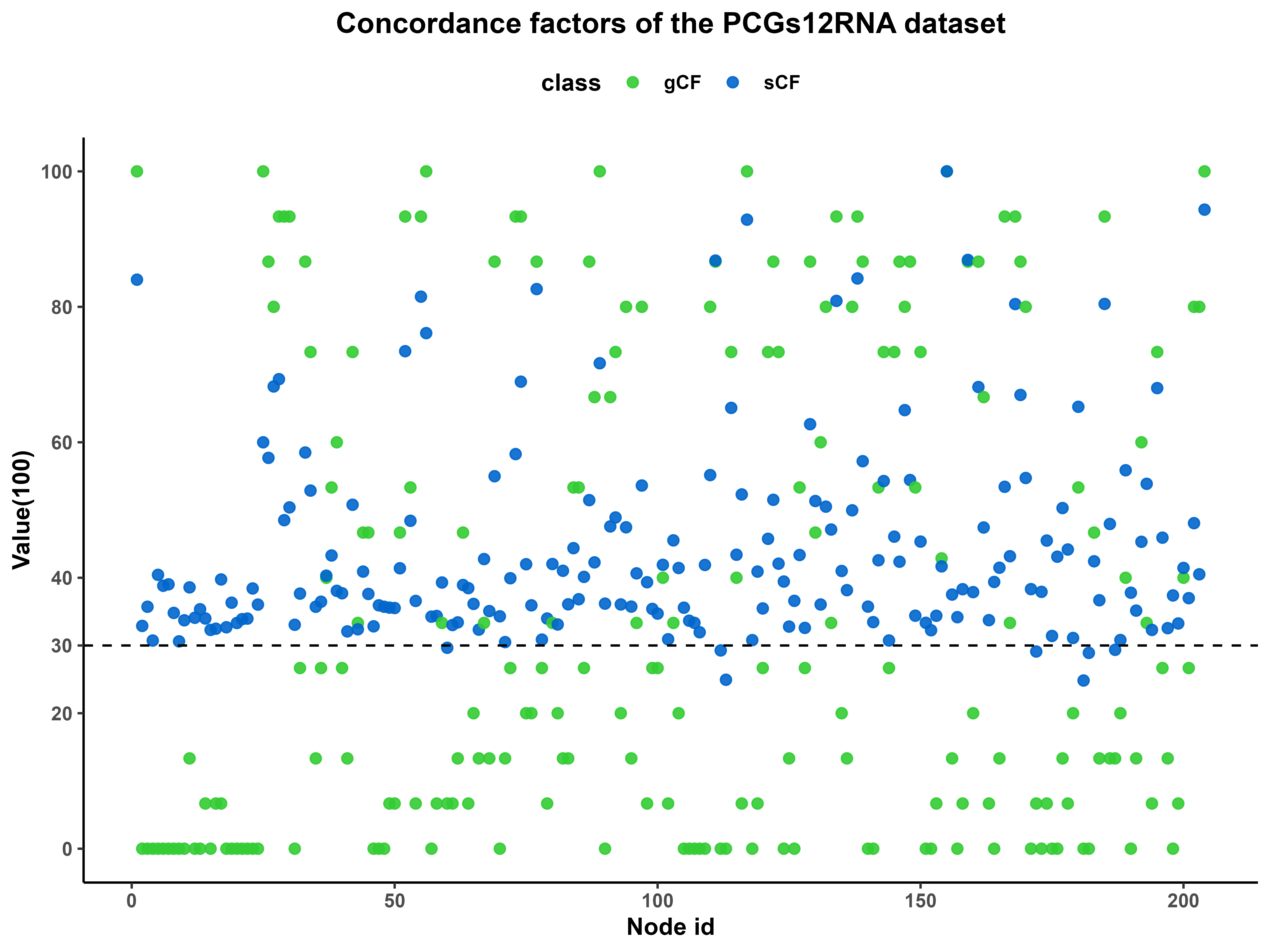

Supplement: Supplementary file 1 [file ijms-26-07222-s001.zip › Figure. S7 concord_PCGs12RNA_plot.png]
